# Tobacco control in complex emergencies: Policy case study of Ukraine

**DOI:** 10.18332/tpc/203838

**Published:** 2025-05-16

**Authors:** Andrii Skipalskyi, Jarno Habicht, Angela Ciobanu, Yelena Tarasenko, Tetyana Skapa

**Affiliations:** 1 World Health Organization, Country Office, Kyiv, Ukraine; 2 Shupik National Healthcare University, Ministry of Health, Kyiv, Ukraine; 3 World Health Organization, Regional Office for Europe, Copenhagen, Denmark; 4 Jiann-Ping Hsu College of Public Health, Georgia Southern University, Department of Biostatistics, Epidemiology and Environmental Health Sciences, Statesboro, United States; 5 Department of Public Health, Ministry of Health, Kyiv, Ukraine

**Keywords:** FCTC, TPD, tobacco control, Ukraine, emergency, NCDS

## Abstract

This policy case study evaluates Ukraine’s implementation of tobacco control measures, using guidance from the World Health Organization Framework Convention on Tobacco Control (WHO FCTC) and its COP8 decision during the ongoing Russian invasion. The study assesses Ukraine’s efforts across three pillars: 1) surveillance, 2) legislation and public health gains, and 3) adherence to the WHO FCTC Article 5.3. Despite war-related disruptions and a humanitarian crisis, Ukraine has upheld and strengthened policies like tobacco taxation, new pictorial health warnings on cigarette packs, advertising bans, and smoking restrictions, largely due to strong policy leadership and international collaborations driven by the European Union integration. Successes and political leadership over the past decade have reinforced Ukraine’s compliance with the WHO FCTC. However, continued threats from the tobacco industry, especially efforts to weaken legislation and obstruct enforcement of regulations on new and emerging nicotine and tobacco products, remain a serious concern. This analysis underscores the vital role of a resilient public health infrastructure and sustained international support in protecting tobacco control progress, particularly during times of crisis.

## INTRODUCTION

The decision made at the 8th Session of the Conference of the Parties (COP8) to the World Health Organization Framework Convention on Tobacco Control (WHO FCTC) in 2018 emphasized the need for countries to uphold treaty obligations during emergencies, reinforcing the global commitments to tobacco control and public health^1,2^.

Post-COP8, the global public health landscape has faced a ‘permacrisis’ marked by overlapping challenges like pandemics, climate change, and conflicts^3^. Such emergencies increase the risk of non-communicable diseases (NCDs) by worsening dietary habits, reducing physical activity, and increasing reliance on addictive substances, including tobacco^4^. In response, the WHO has called for international consultations to address NCDs in emergency contexts^5^.

Since 24 February 2022, Ukraine has faced significant socio-economic disruptions following Russia’s military invasion, which triggered a humanitarian crisis and adversely affected public health^6^. Although tobacco use may seem less urgent amid immediate health system demands, its long-term impact on population health and healthcare resources continues to grow^7^. This case study examined the implementation of Ukraine’s tobacco control policies under the FCTC COP8(20) decision, focusing on three pillars: 1) surveillance, 2) legislation and public health gains, and 3) protection of policies from tobacco industry interference^8^.

## COMMENTARY

Ukraine ratified the WHO FCTC in 2006 and progressively implemented measures to address high tobacco use prevalence over the following decade. In 2017, the second round of the Global Adult Tobacco Survey (GATS) showed a 20% relative decline in current tobacco smoking, reflecting the impact of the comprehensive legislation changes since 2012^9^. A comparative analysis of the Global Youth Tobacco Survey (GYTS) from 2005 and 2017 also revealed a significant drop – over 40% – in youth tobacco smoking rates^10,11^. In 2016, the Association Agreement with the European Union (EU) and Ukraine came into force, mandating the alignment of Ukrainian legislation with EU directives, including the Tobacco Products Directive (TPD)^12^. This political commitment was further catalyzed by the broader healthcare system reform initiated in 2017^13^. However, comprehensive tobacco control legislation was not passed until December 2021, with the law signed in January 2022 and four-stage implementation planned through 2024^14^. Despite the national crisis caused by the Russian invasion, the Ukrainian Government maintained core functions and focused on a dual-track approach, prioritizing resilience and reforms. This approach enabled continued progress in tobacco control and laid the groundwork for this case study, which covers the period of 2022–2024. The following three sections describe the main pillars of the COP8 decision^8^.

### Surveillance

This first pillar of tobacco control emphasizes monitoring tobacco use and evaluating the effectiveness of prevention policies to inform evidence-based responses.

Ukraine continues to collaborate with the WHO and other international partners to ensure ongoing data collection on tobacco use and other NCD risk factors. Although the third round of the GATS was suspended, the following surveillance activities are underway: two rounds of tobacco surveys among adult population in 2023 and 2024 using amended GATS questionnaire^15^; the fourth round of the GYTS in 2023^10^; the sixth round of the Health Behavior in School-Aged Children Survey (HBSC)^16^; and the second round of the WHO STEPwise Approach to NCD Surveillance (STEPS), planned for the coming years^17^.

These surveys will provide critical data to triangulate trends in tobacco and nicotine product use across age groups and establish a baseline for future research on the effects of complex emergencies.

### Legislation and public health gains

The second pillar of tobacco control involves safeguarding tobacco control legislation, enforcement infrastructure, cessation services, awareness campaigns, health workforce training, and sharing information on illicit tobacco trade.

*Safeguarding existing tobacco control laws*

Between 2022 and 2024, collaborative efforts among the government and its institutions, including the Ministry of Health of Ukraine (MoH), intergovernmental organizations, donor agencies, and non-governmental organizations (NGOs), have sustained a comprehensive national tobacco control framework. Key legislative measures include new pictorial health warnings on cigarette packages; stricter regulatory measures on e-cigarettes and heated tobacco products, including a comprehensive ban on advertising, promotion, and sponsorship (with product display at point-of-sale as the sole exception); restrictions on nicotine content in e-cigarettes and ban on characterizing flavors; mandatory reporting of product content; and a comprehensive smoking ban in all public spaces that covers all products. Ukraine has also upheld its effective tobacco taxation policies, aligned with EU harmonization goals^18,19^. A new 2024 tax initiative aims to reach the EU’s minimum tobacco tax threshold in three years^20^.

*Infrastructure for tobacco control*

Ukraine has built a robust tobacco control infrastructure supporting stronger legislation, bylaws, and enforcement. Key components of this infrastructure include strong political commitment and leadership of the MoH, bolstered by social investments made over the past decade. This leadership has enhanced the country’s technical capacity and fostered stakeholder collaboration. Despite ongoing interference from the tobacco industry, this infrastructure has sustained tobacco control efforts, resulting in measurable public health gains, and empowered the government to expand initiatives targeting additional risk factors, like trans fats^21^. The MoH has designated a focal point within its NCD unit to oversee the development of tobacco control bylaws and coordinate the adoption of implementation acts. In May 2024, the MoH was recognized with the World No Tobacco Day Award for its efforts to advance tobacco control measures^22^.

The State Service of Ukraine on Food Safety and Consumer Protection is responsible for enforcing new tobacco control legislation across 25 oblasts (administrative regions). To ensure continued enforcement during martial law, the MoH issued an order exempting tobacco control inspections from the government’s moratorium on business inspections. While inspections related to smoke-free regulations and advertising bans have been limited, restaurant owners and retailers often challenge them in court.

The Ukrainian Public Health Center (UPHC), the country’s primary technical public health agency, has a dedicated tobacco control team within its NCD unit, managing awareness campaigns and cessation programs.

The newly established Centers for Disease Control and Prevention (CDC) network, guided by the public health system law, supports tobacco control through health promotion, local policy development, and monitoring activities^23^.

International support has provided political and technical grounds for Ukraine’s tobacco control efforts, contributing to legislative development and capacity-building. For example, the WHO has supported key programmatic activities through its Biennial Collaborative Agreement with Ukraine, and its long-term commitment is outlined in the Country Cooperation Strategy^24,25^.

Non-governmental and donor organizations have supported tobacco control in Ukraine for over a decade, building a strong advocacy network and NGO ecosystem pivotal for countering tobacco industry interference. Key contributors include the Campaign for Tobacco-Free Kids, Vital Strategies, the Union, and the EU.

Political leadership has also played a critical role. Alongside the MoH, several Members of Parliament have been instrumental in advancing tobacco control legislation, showcasing the value of personal leadership and cross-sectoral collaboration^22,26^.

*Cessation services*

Access to smoking cessation support has been one of the least developed areas of Ukraine’s tobacco control efforts under the WHO FCTC Article 14. However, the MoH has initiated steps to expand cessation services amid the ongoing crisis. Newly introduced pictorial health warnings on cigarette packs include mandatory QR codes linked to a government-run smoking cessation website^27^. Online training courses were made available to family doctors to help them deliver brief tobacco cessation interventions as part of routine care^28^.

*Awareness campaigns*

Over the past two years, the MoH, in collaboration with NGOs, UPHC, and WHO, has supported multiple awareness campaigns on tobacco and nicotine use risks. These initiatives have been vital in educating the public and reinforcing the government’s tobacco control efforts during wartime.

*Training of the health workforce on tobacco control issues*

International partners have supported training of the CDC staff on key risk factors, including tobacco use. WHO also trained enforcement inspectors from all oblasts, focusing on legal support and local implementation^29^.

*Sharing information on illicit tobacco trade to the Convention Secretariat*

Although Ukraine has not yet ratified the Protocol to Eliminate Illicit Trade in Tobacco Products, the MoH continues to submit biannual reports with data on illicit trade to the Secretariat.

### Adherence to the WHO FCTC Article 5.3

The third pillar of tobacco control involves protecting public health policies from tobacco industry interference. Lobbying by the tobacco industry has been a persistent challenge, continuing even during wartime. Several Members of Parliament made unsuccessful attempts to weaken smoke-free laws, while the tobacco industry pushed for favourable tax treatment of heated tobacco products (HTPs)^20^. The government largely resisted these efforts, most notably by designating two major companies – PMI and JTI – as war sponsors^30^. This significantly reduced their influence during the first year of the war despite the continued supply of tobacco products to Ukraine. Ukraine’s efforts were recognized globally, markedly improving its ranking in the Global Tobacco Industry Interference Index from 52nd in 2021 to 16th in 2023^31^.

## CONCLUSIONS

The Russian invasion of Ukraine has triggered significant crises with global repercussions, requiring coordinated efforts, resources, and innovative approaches. Ukraine’s unique ‘response and recovery’ strategy, supported by a coalition of developed democracies, has been vital in preserving tobacco control progress. Focused reforms have helped maintain policies and health gains despite ongoing aggression. Another vital factor is the robust tobacco control infrastructure established over the past decade. The EU integration process and prior tobacco control successes have motivated government and policy actions, while a strong NGO sector has connected institutions, advocacy, and monitoring efforts to counter tobacco industry interference. Ongoing international support remains crucial to sustaining this infrastructure.

**Figure 1:**
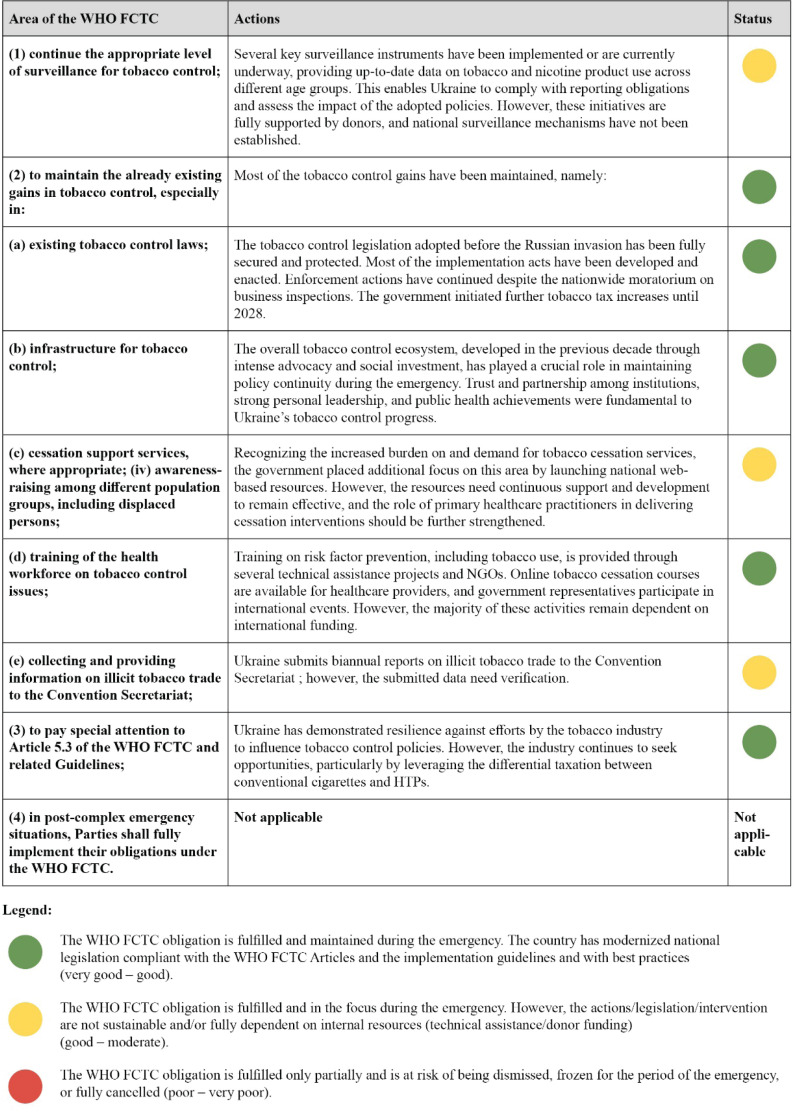
Compliance with the WHO FCTC obligations, based on the FCTC COP8(20) decision ‘Tobacco control in complex emergency situations’

The policy analysis used the ‘traffic light’ approach, indicating high compliance with tobacco control measures (Figure 1). However, the ongoing emergency presents significant risks to long-term sustainability, and the situation remains fragile. While civilian smoking rates have not increased, tobacco use within the defense sector is a growing concern, highlighting the urgent need to expand cessation services and strengthen the role of primary healthcare. Recent proposals to delay tax increases on HTPs have sparked debate^17^, exploiting the absence of a unified taxation policy across the EU. The broader global security crisis, reflected in Ukraine’s ongoing emergency, may also challenge international consensus on UN-led public health priorities. As COP10 in 2024 did not address tobacco control during emergencies, it is crucial for the WHO FCTC governing body to reaffirm the importance of safeguarding public health during conflict, through principled messaging aligned with international law in the upcoming meeting in 2025.

## Data Availability

Data sharing is not applicable to this article as no new data was created.
